# The trace aminergic system: a gender-sensitive therapeutic target for IBS?

**DOI:** 10.1186/s12929-020-00688-1

**Published:** 2020-09-28

**Authors:** Lesha Pretorius, Carine Smith

**Affiliations:** Department of Physiological Sciences, Stellenbosch University, Stellenbosch Private Bag X1, Stellenbosch, 7062 South Africa

**Keywords:** Trace amine, Gut microbiome, Inflammation, Oxidative stress, Drug discovery

## Abstract

Due to a lack of specific or sensitive biomarkers, drug discovery advances have been limited for individuals suffering from irritable bowel syndrome (IBS). While current therapies provide symptomatic relief, inflammation itself is relatively neglected, despite the presence of chronic immune activation and innate immune system dysfunction. Moreover, considering the microgenderome concept, gender is a significant aetiological risk factor. We believe that we have pinpointed a “missing link” that connects gender, dysbiosis, diet, and inflammation in the context of IBS, which may be manipulated as therapeutic target. The trace aminergic system is conveniently positioned at the interface of the gut microbiome, dietary nutrients and by-products, and mucosal immunity. Almost all leukocyte populations express trace amine associated receptors and significant amounts of trace amines originate from both food and the gut microbiota. Additionally, although IBS-specific data are sparse, existing data supports an interpretation in favour of a gender dependence in trace aminergic signalling. As such, trace aminergic signalling may be altered by fluctuations of especially female reproductive hormones. Utilizing a multidisciplinary approach, this review discusses potential mechanisms of actions, which include hyperreactivity of the immune system and aberrant serotonin signalling, and links outcomes to the symptomology clinically prevalent in IBS. Taken together, it is feasible that the additional level of regulation by the trace aminergic system in IBS has been overlooked, until now. As such, we suggest that components of the trace aminergic system be considered targets for future therapeutic action, with the specific focus of reducing oxidative stress and inflammation.

## Introduction

Irritable bowel disease (IBS) is a functional gastrointestinal disorder, which is prevalent in more than 10% of the global population [72]. Although IBS is easily recognised (recurrent abdominal pain associated with change in stool consistency and frequency, as is the case in inflammatory bowel disorder (IBD) [16], but in the absence of structural abnormalities [121]), there is still a lack of any sensitive or specific biomarkers [194], limiting advancement in terms of drug discovery for treatment of this debilitating condition.

It is important to note that epidemiological studies have consistently shown female predominance for IBS in both non-patient and patient populations, with ratios of up to 5:1 in tertiary care settings [157]. Furthermore, many women with IBS report gastrointestinal (GI) symptom fluctuation and exaggeration (flares) during pre-menses and pre-menopausal phases [96, 109, 230]. Although, some of these symptoms seem to be common among non-IBS or normally asymptomatic women too [23, 157]. However, the fact that IBS symptom onset often coincides with gonadal maturation, again implicates hormone levels as confounder in IBS aetiology [97].

In terms of current IBS therapy, treatment strategies range from microbiota-based therapies (probiotics, prebiotics, synbiotics, non-absorbable antibiotics and faecal microbiota transplants) to opioid receptor agonist/antagnoists, and dietary interventions, most of which provide symptomatic relief [45]. Of specific interest to this review, present-day strategies seem focused on addressing clinically evident symptoms only, with relative neglect of inflammation, despite the fact that chronic immune activation and innate immune system dysfunction is implicated in IBS pathogenesis [123]. The importance of considering inflammation in IBS, is underlined by several factors. Firstly, a recent review concluded that gender-differences in inflammation—specifically the fact that prognosis in chronic inflammatory conditions are worse in females, in line with the female predominance in IBS—cannot be fully accounted for by hormonal differences between genders [38]. Secondly, psychosocial stress is the most generally recognized risk factor for both development and relapse of IBS [222]. Here again, females are more at risk, with a significant female predominance reported for anxiety and depression-associated disorders [3]. Taken together, it is clear that gender is a significant role player in IBS risk, but that hormone differences alone is probably not the only predictor of outcome.

We believe that we have identified a “missing link” that ties together gender, diet, inflammation and anxiety in the context of IBS, which may be exploited as therapeutic target. The trace aminergic system was first described in non-human mammals, as having a “sexual cue” function. Most trace amine associated receptors associate closely with olfactory neurons, suggesting a pheromone-type function. In line with this, significant gender differences were reported for trace amine levels [126]. Furthermore—and specifically relevant to the review topic and IBS—trace amine synthesis has been reported in human neurons, where it is thought to modulate neuronal signalling [17, 148, 177], and high levels of trace amines have been found in specific foods, as well as bacterial secretomes [13, 129, 153, 201]. Taken together, it is therefore possible that the trace aminergic system may be an additional level of control/maladaptation in IBS that has been largely overlooked until now. It is also clear that in order to make advances in terms of therapeutic strategies, or even better understanding of disease aetiology, a multi-disciplinary approach is required.

This review therefore aims to provide an integrated and holistic picture of IBS aetiology, including a critical assessment of current methodologies employed in this context where relevant. Drawing from different disciplines in science, we then provide a comprehensive review of the literature on the trace aminergic system, in support of our hypothesis that this system may be targeted therapeutically in the context of IBS.

## The complexity that is IBS

Given the difficulty of IBS management, it would be foolish to underestimate the complexity of the disease aetiology. For the purpose of the current discussion, in the next few sections, we provide an overview of only the most relevant processes at play.

### Oxidative stress and inflammation

Inflammation and oxidative stress go hand in hand, especially in chronic inflammatory disorders, where the poorer prognosis in females has been specifically linked to greater oxidative damage resulting from inflammation [1, 76]. While the susceptibility of cells to oxidative stress is largely variable between individuals and specific tissue types, a review by Jones et al. [106] explains that the GI tract (GIT) is a particularly high reactive oxygen species environment. Furthermore, in cancer literature, the presence of estrogen receptors (ERs) are commonly known to render cells more sensitive to oxidative stress via diminished antioxidant activities [22, 150]. Similar results have been observed in IBS patients. For example, in a study including 36 IBS patients, plasma activities of xanthine oxidase and adenosine deaminase, and plasma concentrations of malondialdehyde (MDA) and nitric oxide, were significantly higher in patients than controls, while superoxide dismutase, catalase and glutathione peroxidase activities were significantly lower [147]. These results suggest that altered oxidant-antioxidant responses are prevalent in patients with IBS. While both males and females formed part of the study, no analysis in gender differences was performed. Furthermore, increased oxidative stress-related markers (elevated MDA, decreased total antioxidant capacity) were reported in 90 IBS patients more recently [44], with a tendency for female patients to have a worse redox profile [43]. Together, these data suggest oxidative damage to be a major contributor to female predominance of IBS.

Of course, plasma redox status is not necessarily an accurate indication of the status at tissue level. Although clinical evidence of oxidative stress within the intestinal wall is lacking, studies in rodent models of IBS have reported evidence suggestive of oxidative stress in the intestinal wall as well. For example, the total antioxidant capacity (measured by FRAP) of large intestine homogenates of rats (IBS induced by restraint-stress) was significantly reduced compared to controls [156, 244]. Moreover, Mozaffari et al. [156] reported significant increases myeloperoxidase activity and lipid peroxidation in the same homogenates. These reductions in anti-oxidant capacity reportedly correlated with gastrointestinal symptomology as well. While similar studies in humans are lacking, it has been reported that neutrophil counts in colonic biopsies of patients with IBS are significantly increased compared to control [39], which may in turn result in increased myeloperoxidase levels, for example, in the colon tissue. Additionally, a very recent study utilising confocal laser endomicroscopy, reported that patients with IBS have a six-fold higher prevalence of colorectal mucosa micro-inflammation than healthy controls [193]. Considering that inflammation and oxidative stress are linked, often occurring in tandem, it is likely that local effects of oxidative damage/stress are implicated in IBS-related gastrointestinal symptomology.

As is the case for many chronic inflammatory diseases, it is difficult to know whether oxidative stress or inflammation manifests first. However, given the self-perpetuating cycle of oxidative stress and inflammation, it is not unexpected that a pro-inflammatory phenotype (increased TNFα and IL-17, decreased IL-10) is prevalent in IBS. Also, in the context of inflammation, a female bias has been reported, with females exhibiting higher inflammatory capacity and generally having poorer prognosis in chronic inflammatory disease [38].

Inflammation is, however, not just an outcome of oxidative stress, but is interconnected with other significant role players in IBS, as demonstrated in the following sections.

### Serotonin dysregulation

Serotonin—or 5-hydroxytryptamine (5-HT)—is a well-known neurotransmitter and neuro-hormone, which modulates several GI functions, such as motility, visceral sensitivity, immune function and blood flow [113]. Additionally, due to its prominent role in the gut–brain axis, perturbations in 5-HT signalling have also been implicated in the pathophysiology of IBS [35, 45, 54, 84, 85, 170, 172]. Mucosal serotonergic enterochromaffin (EC) cells are sensory transducers that respond to luminal stimuli by secreting 5-HT into the intestinal wall to stimulate the primary afferent nerve fibers of the enteric nervous system [15, 134]. Although relatively rare (less than 1% of intestinal epithelia), EC cells produce more than 90% of the body’s 5-HT and have been suggested to affect a variety of physiological and pathophysiological states [84, 142].

Indeed, in IBS, evidence of dysregulated serotonergic signalling has been established. The most reproducible results indicate that patients with diarrhoea-predominant IBS have higher blood levels of 5-HT [14], while patients with constipation-predominant IBS have lower blood levels of 5-HT [6, 68] compared to healthy controls.

Also here, a gender-dependence is evident: 17β-estradiol (E2) regulates the concentration of 5-HT via two mechanisms. Firstly, E2 increases synthesis of tryptophan hydroxylase [20, 21], which is the rate-limiting factor in the conversion of tryptophan to 5-HT, thereby increasing the concentration of 5-HT [26, 215]. Secondly, E2 inhibits gene expression of the serotonin reuptake transporter (SERT), and also acts as a SERT antagonist, consequently promoting the actions of 5-HT by increasing its availability in synapses and interstitial spaces [168, 175]. Beyond increasing concentration and availability of 5-HT, E2 also modulates the actions of 5-HT. This is because the activation of E2 receptors affects the state and distribution of 5-HT receptors. For example, higher levels of E2 in the presence of progesterone (Prog), upregulates ERβ—resulting in upregulation of the 5-HT_2A_ receptor [169] [117, 155]—and downregulates ERα [41]—resulting in a decreased NFκB-associated activation of 5-HT_1A_ receptors [234]. Therefore, during the reproductive phase of a female lifespan (higher E2 and Prog levels), E2 causes an increase in the density and binding of the 5-HT_2A_ receptor.

It is notable that the 5-HT_2A_ receptor gene is expressed in the brain and the gut [187], and has been reported as the main 5-HT receptor in the perception of pain [219], which may contribute to female bias in pain processing, specifically in an IBS context [146]. Interestingly, a study by Pata et al. [172] implicated 5-HT_2A_ receptor gene polymorphisms as a genetic component of IBS pathophysiology. Specifically, a high incidence of homozygous C allele of the 102 T/C polymorphism (also reported in patients with depression and anxiety) and homozygote A allele of the − 1438 G/A promoter region was reported in patients with IBS. Moreover, the patients with T/T genotype had a significantly higher visual analogue score (determines severity of chronic abdominal pain) than patients with other genotypes, suggesting that the T/T genotype potentates pain perception, although it is not unique to IBS. It remains to be elucidated if a gender bias exists for this type of mutation. Nevertheless, in line with this data, abdominal pain is a hallmark of IBS and is often a result of colonic distension and visceral hypersensitivity [53]. Of further relevance, it has been reported that 5HT_2A_−/− mice had smaller enterocytes, fewer paneth cells, and thinner muscle layers, compared to 5-HT_2A_+/+ littermates [77]. However, since this receptor does not seem to affect colonic transit time, IBS treatments targeting 5-HT receptors have classically focused on 5-HT_3_ (facilitates enteric to central nervous system signalling and promotes gut motility), 5-HT_4_ (augments peristalsis and intestinal secretion), 5-HT_1B_ (initiates peristalsis) receptors [73] and even 5-HT_1A_R—for which decreased activity has been linked to exacerbated symptoms of depression [90], a known co-morbidity in IBS. The role of 5HT_2A_ receptors in the context of IBS-related pain remains to be fully elucidated.

### Altered colonic ion secretion

While colonic ion secretion is critically important in maintaining GI motility, there is no concrete evidence that patients with IBS (regardless of the subtype) suffer from primary secretory diathesis [34]. Moreover, it is reasonable to suggest that different IBS subtypes would be characterised by different secretory ion profiles, resulting in either constipation (more common in females) or diarrhoea (more common in males). In terms of a reproductive hormone effect, E2 is a known modulator of ion-secretion, also independent of its effect on 5-HT signalling. Both ERα and ERβ have been detected in distal colonic crypts [218], where E2 was shown to inhibit epithelial chloride ion secretion in female rats [48], resulting in significant water and salt retention during high estrogen states [165]. Of interest, the gender bias for the anti-secretory action of estrogen was attributed to gender specificity of ion transporter protein expression profiles [48]. Furthermore, E2 reduced currents mediated by the KCNQ1:KCNE3 potassium channel in an Ussing chamber model [4]. Similarly, more recent data shows that E2 links to intracellular calcium, cystic fibrosis transmembrane conductance regulator and Cl^−^/HCO_3_^−^ secretion [239]. Seeing that E2 inhibits colonic chloride ion secretion (consequentially reducing water movement to the lumen), it makes sense that females with IBS generally present with reduced colonic transit/GI motility and constipation, symptoms which alter drastically during menses.

### Gut dysbiosis

Up to now, gender-association has been a continuous thread through all factors contributing to IBS aetiology. However, although it is clear in other disease contexts, e.g. auto-immune disease, that a gender bias indeed also exist in terms of gut microbial content and/or function [191], this association is less clear in IBS, due to a relative lack of research in this context.

Nevertheless, the gut microbiota is widely regarded as a regulatory system that actively mediates numerous physiological functions as part of its symbiotic relationship with its host, via generation of metabolites to affect both nearby and distant organs [137, 231], including the brain [139]. This ability to predict clinical phenotype was even recently suggested to be superior to the predictive power of genetics [197]. Indeed, altered bacterial composition, the so-called dysbiosis, is associated with a spectrum of diseases, including neuropathology and inflammatory conditions [64]. For example, germ-free animals demonstrate delayed gastric emptying and intestinal transit, reduced migrating motor complex cycling and propagation, and reduced GABA and VAP-33 gene expression, when compared with animals raised in a normal laboratory environment [11]. Thus, it is not surprising that some form of compositional dysbiosis (altered microbial alpha and/or beta diversity) has been implicated as an etiological factor in the development of various gastrointestinal disorders, including IBS [114, 159, 171, 185], where it is thought to drive persistent low-grade inflammation and chronic gut dysfunction [47]. Globally, data on IBS patients suggest reductions in microbial diversity, altered proportions of specific bacterial groups, shifts between mucosal and luminal bacterial abundance [36], and a higher degree of temporal instability of microbiota [64], when compared to healthy individuals.

However, there are several shortcomings of exploring compositional dysbiosis in the context of IBS. Firstly, the compositional alterations reported in the literature are not specific to IBS—similar changes in microbial diversity are found in numerous diseases and conditions [47, 64]. This raises questions regarding their specificity as potential disease biomarkers. Similarly, some authors have criticized whether a relative microbial imbalance that is assessed in a cross-sectional approach, would accurately represent a disease or reflect whether this imbalance occurs secondary to disease-related behaviours (poor lifestyle choices), as this requires analysis of consecutive samples with simultaneous disease variation [71]. Secondly, many of these studies have produced conflicting results within the IBS population [66] for various reasons commonly ascribed to methodology or study design [130, 140, 184, 217]. Thirdly, an improvement in the fairly basic genus-species analysis most often employed [194] is required before significant advances in knowledge gain on the topic are made. This is corroborated by probiotic-focused studies that have emphasized strain-specificity in bacterial function [86], let alone species specificity. Fourthly, the profile of a ‘normal’ or healthy microbiota is unknown. In our opinion, and those of several other authors, it is impossible to define a healthy microbiome due to the high degree of inter-individual variation [50, 58], cultural habitual dietary habits and other population-specific factors. This adds to the complexity of selecting suitable controls for gut microbiome studies. Accordingly, extreme care to choose controls similar not only in age and gender, but also cultural background and nutritional habits, is vital to the reliability of any study (but may at the same time also limit its broader relevance). Lastly, efforts to standardize testing, with the introduction of the ‘dysbiosis index’ [37] has thus far been unsuccessful, since the predictive value of the index was reportedly low and non-specific [71]. Given the fact that compositional analysis does not seem to be sufficiently sophisticated yet to lead to therapeutic advancement in medicine, another option is to rather consider functional effects of the total microbiome secretome (i.e. the secretory products of all microbes present in the gut) on its host.

Indeed, another grouping of researchers tends towards analysing *functional* dysbiosis, or the alterations of the microbial secretomes, in diseased states [59, 104, 192, 242, 243, 247]. This is achieved through mass spectrometry or nuclear magnetic resonance techniques, and allows researchers to associate the absence or presence of certain microbial-associated molecules to disease symptomology [224], which may provide an avenue for discovery of disease-specific biomarkers or novel targets for therapeutic treatment [5]. Given the fact that at least some microbial secretory products are competitive survival tactics, the relative absence of a “competing species” may alter secretome content (and thus effect on host cells) significantly, but linearity of secretome change to bacterial predominance cannot be assumed, lending further support for favouring analysis of functional dysbiosis.

In the context of microbiome-associated functional effects, recent studies seem to highlight metabolites derived from microbial transformation of dietary components as having significant effects on several physiological processes [115, 116, 198, 246]. One of the first studies to explore microbial metabolism in the IBS context reported that these patients had increased production of hydrogen gas (H_2_) (fasted breath test) [118]. The authors suggest that the difference in H_2_ production may be associated with small intestinal bacterial overgrowth (SIBO), since SIBO is common among IBS patients [87] and is associated with higher levels of H_2_ production in fasted states [112]. Additionally, altered proportions of specific bacterial species, such as decreased *Lactobacilli* in IBS patients, may alter the amount/distribution of the by-products of microbial metabolism (H_2_). Indeed, *Lactobacilli* are less gas producing than some other bacteria, such as *Clostridia* and *Enterobacteriaceae* [162, 166]. This is confirmed by another study in which colonization by *Clostridium* spp. was associated with excess gas production, abdominal discomfort and bloating among IBS patients [204]. As such, the relative dysbiosis of IBS patients may result in higher levels of excreted H_2_ as a functional consequence. Indeed, altered microbial fermentation of carbohydrates results in the excessive production of H_2_ and methane gases, the elimination of which is essential to maintain efficient fermentation in the gut.

While H_2_ only represents a single by-product of microbial metabolism, these findings demonstrate the potential of microbial-derived metabolites to alter host functioning. The most obvious example of this lies in the relatively successful use of probiotics as a treatment option for individuals with IBS. Probiotics are “live strains of strictly selected microorganisms which, when administered in adequate amounts, confer a health benefit on the host” [99]. Theoretically, probiotics, primarily those containing *Lactobacillus* and *Bifidobacterium* spp., should beneficially modulate the gut microbiota through production of antimicrobial proteins, which should reduce pathogenic bacteria and interfere with epithelial adhesion [62, 78, 144, 210], among several other mechanisms of action [64]. In a meta-analysis including 35 RCTs, probiotics were shown to have beneficial effects with regard to abdominal pain, bloating and flatulence scores in IBS patients [78]. Additionally, authors described superiority of multispecies probiotics to single species probiotics, but found no specific combination of multispecies probiotics predominant to another. As such, clarification with regards to which combinations of species are effective in treating specific IBS subtypes, and optimum treatment dosages and durations are still required [124]. Thus, not enough is known for compounding of probiotics into treatment formulations, or to accurately prescribe probiotic strategies.

**Fig. 1:**
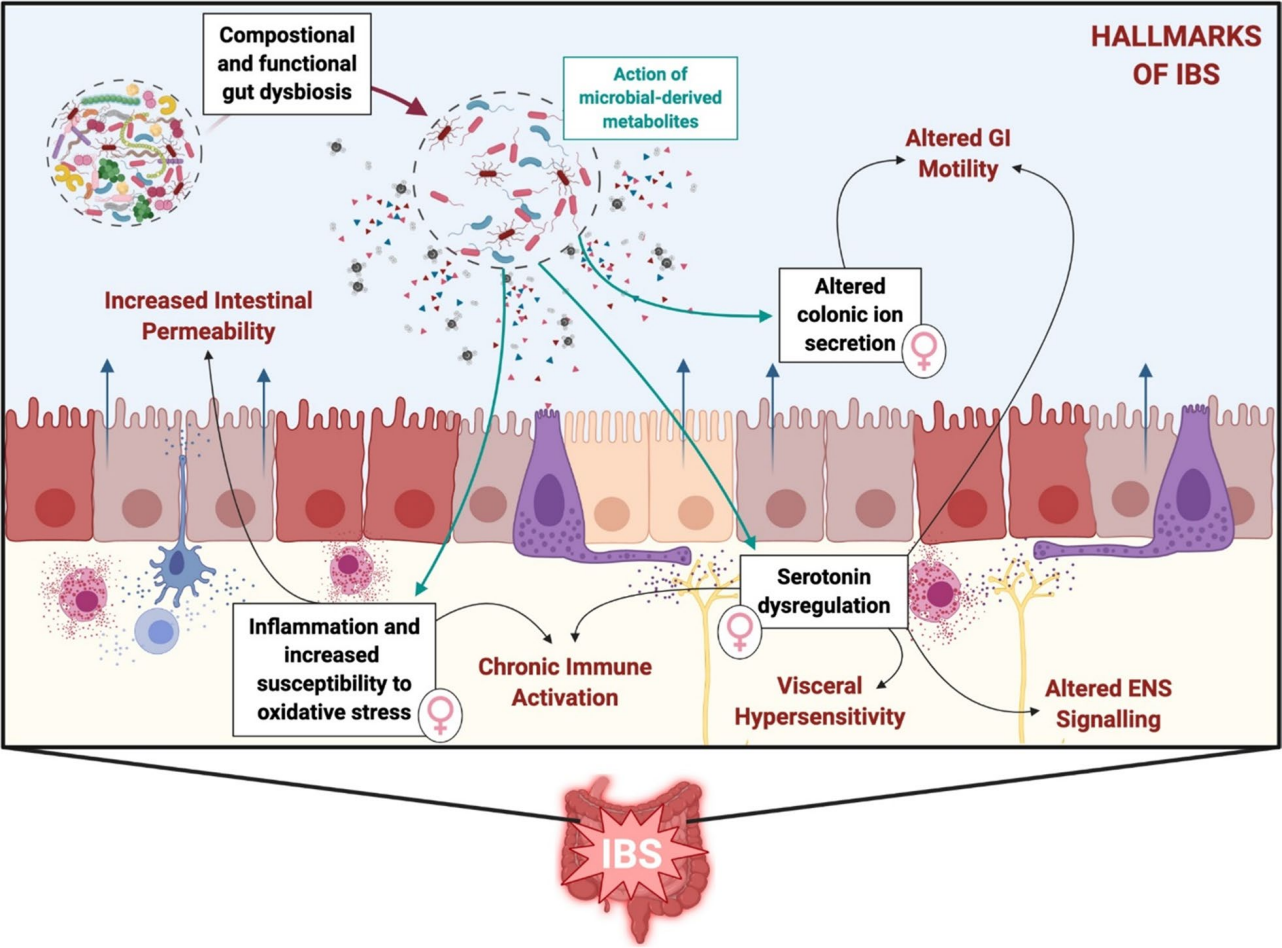
A simplified visualisation of IBS-associated pathology. *GI* gastrointestinal, *EC* enterochromaffin, *5-HT* serotonin

An integrated presentation of IBS-associated pathology is also presented visually in Fig. 1. Taken together, the female bias towards exacerbation of IBS symptomology is clear, as well as its prominent links to the actions of microbial-derived metabolites, or as we suggest, the trace aminergic system.We believe that trace amines (which are, at least in part, by-products of microbial metabolism) may provide the molecular link to explain the association between gut microbiome dysbiosis, IBS, inflammation and central nervous system conditions such as depression and anxiety (both high incidence co-morbidities in IBS).

## The trace aminergic system

Trace amines (TA) are a class of biogenic amines produced endogenously in humans, but also present in bacterial secretomes and certain foods. Common TA include β-phenylethylamine (PEA), tryptamine (TRP) and ρ-tyramine (TYR), which are derived from their respective amino acid precursors l-phenylalanine, l-tryptophan and l-tyrosine. Synthesis of TA primarily occurs through the enzymatic action of aromatic l-amino acid decarboxylases (AADC) [17]. While endogenous synthesis of TA is often reported to be neuronal, AADC are also present in non-neuronal tissues, including the epithelium of the GI tract [122, 226]. Additional sources of TA include those derived from food and microbes.

Trace amine-associated receptor (TAAR) 1 is the most thoroughly studied of the receptors in humans and has both central (acts as a rheostat of dopaminergic, glutamatergic, and serotonergic neurotransmission) and peripheral (regulates nutrient-induced hormone secretion and immune responses) roles [80]. In the next few sections, we review different aspects of the trace aminergic system, as it relates to IBS.

### TA “toxicity” risk

Interestingly, foods containing high levels of TA, such as cheese, wine, sausages and other fermented foods—all commonly linked to exacerbated symptoms in IBS—are foodstuffs largely dependent on bacterial fermentation [107, 153, 155]. Lactic acid bacteria (LAB) are considered the primary biogenic amine producers in fermented foods. Indeed, various *Lactobacillus* spp. starter cultures have been studied with the aim of mitigating the potential health risks (headaches, heart palpitations, vomiting and diarrhoea) associated with excessive biogenic amines levels [7]. Interestingly, the authors of this study reported that TYR and PEA were produced by 14.4% and 12.4% of LAB isolates (fermented sausages) respectively, all belonging to *L. curvatus* species. As such, the authors recommended that *L. sakie* be used as the predominant LAB in preparation of these sausages in the future. In fact, the inability of a strain to synthesize biogenic amines is included in the selective criterion for malolactic starter cultures [220]. It is, however, important to highlight that the biogenic amine producing ability is a strain-specific characteristic, as variability in aminobiogenetic potential between different strains belonging to the same species is evident [13]. Regardless, the link between TA levels and fermentation is clear. Indeed, the expression (transcriptional induction) and/or activation (catalytic modulation) of LAB amino acid decarboxylation systems is reportedly an adaptive response to energy depletion, and is considered a strategy that counteracts acid stress [180], since decarboxylase activity can lead to membrane energization and increased environmental pH. Moreover, the strain dependent (rather than species specific) presence of decarboxylases genes involved in biogenic amine production eludes to horizontal gene transfer between strains as an adaptive mechanism of survival in specific environments (such as the GIT) [51, 131, 136]. Therefore, these decarboxylation mechanisms represent an important ecological tool, which can confer a competitive advantage in acid or nutritional stress conditions [75, 178, 179, 195].

Of the potential health risks related to biogenic amines, the most severe symptoms are said to be caused by histamine and TYR [13]. Interestingly, a study investigating the self-reported food intolerance of 197 IBS patients, reported that 84% of these individuals recounted symptoms related to at least one of the surveyed foodstuffs, of which, 58% experienced GI symptoms from foods rich in biogenic amines (wine, beer, salami and cheese) [28]. Additionally, histamine-containing food were also considered as causes of IBS-related symptoms. The resultant symptoms are reportedly induced via chemo-stimulation of gut or immune cell receptors [88]. Of note, the authors also reported that females reported more food items causing symptoms than males [28], although no potential explanation was provided. This study, along with several others, emphasises the high perceived food intolerance among IBS patients [57, 152, 211]. Histamine (a weak TAAR1 agonist) is directly involved in inflammation, while TYR intoxication facilitates the “cheese reaction”. This reaction, most commonly described in the context of cheese consumption, is the result of a food-drug interaction, where the food can be any TYR rich food and the drug usually a non-selective monoamine oxidase inhibitor (MAOI). Mechanistically, TYR increases sympathetic responses by indirectly acting as a sympathomimetic (displaces stored norepinephrine (NE)), thereby increasing the levels of circulating NE. The use of MAOI exacerbates this action by inhibiting the metabolism of both TYR and NE. As such, symptoms of the “cheese reaction” include dietary-induced migraine, nausea, vomiting, increased cardiac output, respiratory difficulties and elevated blood glucose levels [135]. This has had important implications in patients using MAOI, in which not even low levels of biogenic amines can be metabolised effectively [200]. MOAI have been prescribed to patients with chronic anxiety to improve 5-HT signaling, and intriguingly, 54 to 94% of treatment-seeking IBS patients will have a co-morbid psychiatric disorder [67, 199] of which, anxiety and depression are the most common. The resultant biogenic amine sensitivity that these patients experienced led to the development of new generation MAOI, so-called reversible MAO-A inhibitors [145].

As mentioned, histamine is indeed a weak TAAR1 agonist [250], and various reports suggest between 5 and > 50 μM are required for the activation of TAAR1 (similar potency as TRP, NE and synephrine) [29, 127, 237]. Even though patients with IBS reportedly have elevated levels of histamine in mucosal supernatants/biopsies, of up to 50 ng/mL mg [32, 91], it is unlikely that these endogenous levels result in TAAR1 activation. However, exogenous or dietary histamine consumption could contribute significantly to the levels of histamine in the gut, contributing to potentially detrimental effects—although most likely independent of TAAR1 activation. For example, certain fish and varieties of cheeses contain up to 2000 mg/kg of histamine, and the ingestion of 75 mg of histamine is reported to cause symptoms of intoxication in healthy individuals [188, 236]. A recent study by del Rio et al. [60] also reported that co-treatment (on HT-29′s) with TYR and histamine was associated with stronger (or synergistic) cytotoxic effects in vitro than treatment with either TYR or histamine alone, an effect achieved in the absence of TAAR1. These results indicate that histamine increases the cytotoxicity of TYR at concentrations prevalent in some foods (levels generally considered safe for consumption) [60].

While the symptoms of the “cheese reaction” and histamine intoxication are not specific to IBS, other biogenic amines may similarly trigger IBS-specific symptoms by promoting visceral hypersensitivity via the action of bioactive mediators and/or luminal distention [10, 55, 238]. As such, other biogenic amines (PEA, putrescine, cadaverine, agmatine and spermidine) can also cause toxicity, but in cases where multiple biogenic amines are present, they are said to potentiate the effects of histamine and TYR by inhibiting their metabolizing enzymes [176]. Taken together, the toxicity of any biogenic amine will depend on the type of amine, the amount of amine, the individual host sensitivity or allergy, and the consumption of MOA inhibitory drugs (or ethanol), which inhibits or reduces the aminooxidase enzymatic systems responsible for the detoxification of exogenous amines [207].

While these health risks are well-researched with regard to food safety and regulation [201], it is concerning that many LAB are commonly used as probiotics. Indeed, another study has raised concern that some *Lactobacillus *rhamnosus strains often used in probiotics may produce biogenic amines [129]. Moreover, not only LAB predominant probiotics should be considered, but probiotics with *Enterococcus*, *Streptococcus*, and *Lactococcus* species may also potentially produce biogenic amines [13]. In fact, decarboxylase activity is often expressed independently of cell viability, since these enzymes maintain activity after cell lysis, even in harsh environmental conditions [82, 120, 154, 196]. As such, it may be premature to advocate probiotic treatment as a blanket supplementation strategy for therapeutic relief of IBS patients, and at least some individualisation is required to increase efficacy and mitigate risks of adverse outcomes. Beyond the importance of investigating the decarboxylation activity of probiotic or functional cultures before their use, in the context of gastrointestinal disease and symptomology, it may also be important to elucidate negative effects (if any), that chronic exposure to low levels of these biogenic amines could cause.

### Microbial-derived TA modulate host functioning

It is proposed that through the production of bioactive metabolites, such as biogenic amines, the gut microbiota may increase an individual’s susceptibility to GI inflammation via modification of intestinal epithelial function and mucosal immune activity [46, 235]. For example, several intestinal microbes synthesize various amino acid decarboxylases, which means that they have the capability to sequester amino acids, convert them into TA, and thereby alter the distribution of metabolites, such as calcium, 5-HT, trimethylamine *N*-oxide (TMAO) and immune cell mediators in the host, as part of the symbiotic relationship between the gut microbiome and host. However, changes in TA metabolism have already been correlated to both inflammation of the bowel [233] and decreased microbiome complexity (dysbiosis) [205], which suggests that in pathological states, this altered TA metabolism may have functional consequences, that may manifest as or promote disease symptomology. Given the strong links of IBS with depression, and that microbial-derived biogenic amines are similar in structure to monoamine neurotransmitters, TA/TAAR1 should be considered as potential biomarkers and/or therapeutic targets. To motivate this point, this section will discuss (i) the significant presence of TA-producing microbes in the gut, (ii) the optimal conditions of a dysbiotic gut for the generation of microbial-derived TA and, finally (iii) an example of functional modulation by trimethylamine (TMA) and TMAO.

Firstly, in intestinal metagenomes of healthy individuals, tryptophan decarboxylase homologs were found to be present in 9% to 17% of individuals, suggesting that microbe-derived TRP may be more prevalent in the gut than previously thought [232]. Indeed, the gut microbiome of IBS patients is often dominated by Firmicutes [105], the phylum from which the majority of the tryptophan decarboxylases derive. In fact, the gut microbiota features a myriad of metabolizing enzymes, such as various decarboxylases, dehalogenases and amine oxidases, which may facilitate the formation of other TAs (e.g. octopamine and synephrine), as well as functionally active TA-metabolites. While no studies have investigated the percentage of TA producing bacteria in IBS populations compared to healthy individuals, these results suggest that the TA-production capacity of the gut microbiome is significant, and has been largely overlooked or underestimated.

Secondly, according to two independent in vitro studies on known microbial producers of TA (*Lactobacillus brevis* CECT 4669, *Enterococcus faecium* BIFI-58 and *E. faecium* EF37), various physiochemical factors influence microbial synthesis of TA [81, 138]. These factors include incubation temperature and time, environmental pH, pyridoxal-5-phosphate supplementation, sodium chloride concentration and most importantly, amino acid substrate availability, most of which are optimal within the human GIT. Of note, some of these factors may be altered in pathological states, towards promoting microbial TA production. For example, luminal pH is reportedly altered in individuals with dysbiosis, and this may contribute to mucosal inflammation and enterocyte dysfunction [24]. With regard to IBS, Ringel-Kulka et al. [190] reported that colonic intraluminal pH levels were significantly lower in IBS patients (all disease subtypes) when compared to controls. Similar findings have also been reported in patients with IBD [164], with one study reporting low colonic luminal pH values (pH 5.3 patients *vs* pH 6.8 controls), which were associated with active disease states [206]. Interestingly, a pH range of 4 to 5.5 is reported to increase amino acid decarboxylase activities and thus enhance TA production [81, 138]. This microbial response is a well-documented adaption to pH/acid stress (as already briefly discussed in “TA “toxicity” risk” section), and suggests that more efficient TA production may occur in individuals with dysbiosis.

Finally, TMA is a selective agonist of TAAR5 [126, 227, 245], and there is no known mammalian pathway for its synthesis. As such, the production of TMA seems to be exclusive to the metabolism of choline, betaine, and carnitine by microbes [52, 108, 248], with the administration of antibiotics to mice, reducing the levels of urinary TMA [79, 240]. Interestingly, raised levels of TMA have been reported to result from dysbiosis at various mucosal sites, such as intestines, in both mice and human models [74, 246]. While increased levels of TMAO are generally associated with extra-intestinal diseases (CVD), TMAO may cause intestinal inflammation and oxidative stress [40]. Considering that TAAR5 expression has been reported for several leukocyte populations [8], particularly B lymphocytes, it is clear that increased levels of either TMA or TMAO could be involved in initiating or perpetuating intestinal inflammation, as is common in IBS. Moreover, altered levels of TMA/TMAO can reportedly alter the growth and secretion of metabolites of several intestinal bacteria [102]. As such, TMA/TMAO not only affects host functioning, but can alter the luminal environment too, perpetuating dysbiosis.

### Mechanism of action of TA in the gut

Perhaps due to a relative lack of cross-disciplinary communication in this context, despite the knowledge of their existence, or perhaps as a result of the bias in favour of compositional, rather than functional assessment of the gut microbiome, data on the specific actions of TA in the human gut are still relatively limited. However, some insights into the function of TA in the context of IBS may be derived by considering their extra-intestinal effects.

### Direct cellular effects of TA

Direct cytotoxic effects of PEA, TRP and TYR on MonoMac-6 and HEK293 cell lines was investigated (MTT assay) [133]. Data showed that 62.5 μg/mL of each TA independently reduced MonoMac-6 viability by 20%, and 125 μg/mL of TRP reduced MonoMac-6 viability by 80%. While the HEK293 cells were more resistant to the cytotoxic effects of the TA, 500 μg/mL of TRP also reduced viability by more than 80%. From this study, the most cytotoxic TA seems to be TRP, while TYR had the least cytotoxic effects. However, the lack of an in vivo context limits the interpretations which can be made by these data. Moreover, the lack of reported absolute concentrations of endogenous TAs in the gut/intestinal mucosa of humans makes it difficult to draw firm conclusions on the physiological relevance of these findings and the specific doses. To “bridge” this gap in literature, we propose the consideration of firstly, the contribution of exogenous TA consumption (in food) to levels in the gut. For example, it has been previously reported that dietary concentrations of PEA and TYR indeed stimulate the gut, altering intestinal blood flow in an ex vivo model [31]. Secondly, we considered that the contribution of TAs derived from major TA-producing microbes in vitro, suggests that the TA range selected for the generation of the WST-1 data is feasible. For example, *Staphylococcus pseudintermedius* ED99 cultured in media containing 2 mg/mL of l-tryptophan, l-phenylalanine and l-tyrosine produces 231 ± 10 μg/mL TRP, 557 ± 8 μg/mL of PEA and 360 ± 9 μg/mL TYR in vitro [133]. These data suggest that in the gut, in the presence of potentially numerous TA-producing microbes, significant concentrations of TAs may be present. Thus, while the endogenous levels of TAs are not known, the exogenous (and potentially endogenous) contribution may be significant enough to suggest physiological relevance.

Moreover, the presence or production of TA is reported to enhance the ability of *Staphylococcus* and *Enterococcus* spp. to adhere to intestinal epithelium, promoting consequential internalization and enterocyte cytokine secretion [75, 133], potentiating colonization as a potential adaptive advantage for these species. Indeed, TA bound to the α2-adrenergic receptor induced cytoskeletal reorganisation, which facilitated host cell colonization to boost adherence of both TA-producing and non-TA-producing bacteria [133]. Interestingly, the addition of 10 mM tyrosine (which resulted in formation of ± 140 nmol TYR) significantly improved bacterial adherence to colon epithelium by threefold, while direct supplementation with 140 μM of TYR did not affect adherence [75]. The authors speculated that the activation of the TYR biosynthetic pathway, rather than the production of TYR, could be involved in the enhancement of microbial adhesion. Nevertheless, this data illustrates the complex mechanisms at play to facilitate TA effects.

Taken together, these results suggest that dose specificity is an important consideration. What is not known, is the range in which microbes potentially benefit from TA (producing or produced), while conferring host cytotoxicity, and what implications this could have for IBS.

In terms of enteroendocrine function, PEA was reported to stimulate gastrin secretion from stomach G cells in a rat model [61]—which in the IBS context is linked to ulceration and dyspepsia [69]. This again points to a direct detrimental effect of TA in the IBS context.

With regard to colonic ion secretion, TRP in particular was reported to promote colonic ion secretion [232], however the nature of this ion secretion and potential preference to a specific ion(s) were not reported and thus, require further investigation before interpretation of the significance of this finding is possible. Nevertheless, this data potentially suggests that TRP-mediated signalling might affect GI transit. Furthermore, from the known effects already outlined for E2, we can postulate that in general E2 and TAs, such as TRP, have opposing effects on colonic ion secretion and GI motility. This could explain an exacerbation of symptomology during menses, when ion secretion and thus GI motility shifts from one side of the spectrum to the other in female patients, an effect that would be heightened in the presence of a high TA (or at least TRP) load. The potential of manipulating TRP levels to achieve optimal GI transit in IBS, warrants TA profiling in IBS.

### Modulation of oxidative stress and inflammation

Intracellular accumulation of Ca^2+^ is commonly associated with oxidative stress, damage and inflammation in various chronic conditions. Of relevance in this context, binding of TA to human TAAR1 results in the influx of Ca^2+^ as a result of activated TAAR1 coupling to Gα_s_ and G_q_ proteins [158]. Upon stimulation of these G-proteins, intracellular messengers such as cAMP and IP_3_ accumulate and activate downstream proteins such as PKB and PKC, which mobilize intracellular Ca^2+^ stores, as well as promote extracellular Ca^2+^ influx [29, 30, 33, 127]. Excessive Ca^2+^ influx may lead to endoplasmic reticulum stress and mitochondrial dysfunction, rendering a cell with an unfavourable redox profile, and thus several regulatory mechanisms intricately control intracellular Ca^2+^ levels. However, in overabundance of TA, regulatory mechanisms may be overwhelmed. In line with this, histamine—a known mediator of inflammation and known to be increased in IBS—has also been reported to increase the intracellular Ca^2+^ response in an EC cell line (P-STS) [181]. Apart from the resultant direct oxidative stress, histamine and TA may also exacerbate aberrant 5-HT signalling linked to IBS symptomology (altered GI motility, visceral hypersensitivity and immune activation). Interestingly, TA may also indirectly cause increased susceptibility to oxidative stress through their modulation of serotonin, as 5-HT_2A_ receptor activity was found to increase intracellular calcium via the mitogen-activated protein kinase pathway [229]. This may cause an intracellular Ca^2+^ burden within the surrounding intestinal tissue, resulting in symptoms such as abdominal pain. As such, it may be important to consider targeting TA availability or modulation of TAAR1 expression in an effort to curb Ca^2+^-associated visceral hypersensitivity in IBS patients with severe abdominal pain.

Another common trace amine, 3-iodothyronamine (T_1_AM), may also contribute to changes in Ca^2+^ homeostasis and alter the pro- and antioxidant balance in the intestine and surrounding tissue by interacting with various receptors (such as α2-adrenergic receptor) [101, 249]. For example, T_1_AM reportedly increased the amount of hydrogen peroxide released by rat liver mitochondria [223]. Interestingly, Chiellini et al. [42] reported that exogenous T_1_AM (and its metabolites) primarily undergo biliary and urinary excretion, and subsequent reports have suggested the presence of significant endogenous levels of T_1_AM in stomach and intestine, at least in mice [100], suggesting that the pro-oxidative effects of T_1_AM may not be limited to the liver. While the precise biosynthesis of T_1_AM in humans remains to be confirmed, Hoefig et al. [100] has demonstrated that the intestine expresses the enzymatic machinery (intestinal deiodinases and ornithine decarboxylase) required for T1AM biosynthesis from thyroxine, while other authors highlight the potential of the gut microbiota to generate T1AM [89, 203]. In terms of relevance to IBS, interactions of T1AM with histaminergic circuitries has been proposed [249], warranting investigation in the context of both inflammation and oxidative stress related symptomology.

In terms of inflammation a relatively recent metabolomics study indicated elevated faecal PEA levels in patients with Crohn disease [103], suggesting a pro-inflammatory effect for trace aminergic signalling. Similarly, abundance of *Faecalibacterium prausnitzii*—a species that has a reported role in mitigating inflammation in the colon [128, 149]—also correlated inversely with PEA levels in IBD patients [205]. Since significant consumption of phenylalanine is associated with growth of *F. prausnitzii* [95], its absence in dysbiotic conditions may increase the availability of phenylalanine to TA-producing microbes, resulting in elevated PEA levels. This potential mechanism should be evaluated more comprehensively in IBS-specific models.

Furthermore, PEA and TYR are chemotactic for polymorphonuclear cells (PMN) [8], major role players in inflammation and in particular, secondary damage to host tissue during inflammation. Considering that low levels of TA are normally present in the GIT—the interface between mucosal immunity and microbes—it is possible that in dysbiosis, the ensuing immune activation and altered microbial behaviour may promote or exacerbate intestinal inflammation. Within the context of IBS, and specifically female-predominance, it is necessary for future experiments to elucidate the contribution of TA to chronic intestinal inflammation, and whether or not female reproductive hormones affect this in any way.

### The role of TAARs

Most TAAR-related research to date was performed in microbiology contexts. Although some neurophysiology investigations have reported on TAAR, this niche is largely unexplored. Generally, the available literature seem to suggest that TA and TAAR are not exclusively “paired”, with TA able to act as ligand for several other receptors. Furthermore, TAARs—in particular TAAR1—seems to be a rate-limiting factor in TA-associated effects, as its presence have been linked to opposite effects than described for TA.

For example, in contrast to the over secretion of gastrin linked to TA, TAAR1 activation by a selective small molecule agonist was associated with elevated plasma levels of peptide tyrosine tyrosine (PYY) and glucagon-like peptide-1 (GLP-1) [186], which may be protective, as decreased levels of secreted PYY and GLP-1 from L-cells are implicated in IBS pathogenesis and symptomology [70, 167]. Similarly, in terms of modulation of leukocyte responses specifically, TAAR1 is differentially expressed in several leukocyte populations, such as PMN, B and T lymphocytes, monocytes, and natural killer cells [8, 56, 160, 214], thus TAAR1 activation may regulate leukocyte differentiation and activation. Indeed, expression of both TAAR1 mRNA and protein components are upregulated in primary human lymphocytes after activation with PMA and PHA [160, 228]. However, one would expect in the context of IBS, that chronic (rather than acute) activation may downregulate TAAR expression overtime. As such, the importance of conducting in*/*ex vivo testing should be emphasized, and certainly warrants future investigations. Furthermore, T helper lymphocyte differentiation toward Th2 phenotype may be regulated by the activation of leukocyte TAAR1 and TAAR2 [8]. However, in the same study, TAAR1 and TAAR2 activation were also reported to mediate IL-4 secretion from T lymphocytes and immunoglobulin E (IgE) secretion from B lymphocytes [8]. Of relevance to the allergy-like symptoms prevalent in IBS, both IL-4 and IgE mediate allergic inflammatory responses [173, 216], inducing mast cells to release histamine upon IgE binding, and are implicated as central role players in IBS [12, 125]. This seemingly dichotomous role for TAAR in the activation of immune cells remain to be further elucidated.

In terms of its effect on serotonergic signalling, highest predominance of TAAR1 is described in neuronal aminergic pathways [18]. Of specific relevance, in a rodent knockout model, the absence of TAAR1 was associated with increased aminergic (dopaminergic and serotonergic) signalling [177], again suggesting a dampening effect for TAAR1. In terms of applicability to this review, IBS-associated 5-HT dysregulation—which is implicated in altered GI motility [132, 221] and visceral hypersensitivity [143]—is already therapeutically targeted by 5-HT_3_R antagonists [85, 141]. Assuming that a higher TA load results in reduced TAAR1 expression (specifically in EC cells) in chronic conditions, a more accurately targeted approach might include modulation of trace aminergic signalling, thereby eliminating the cause of 5-HT dysregulation.

It is important to note, however, that TAAR signalling is additionally complicated by three factors. Firstly, genetic variations in the form of TAAR polymorphisms, as well as their clinical relevance, cannot be excluded [46], since function-altering polymorphisms of TAAR1 [209] and TAAR2 [27] have already been reported. Secondly, TAAR expression is often recorded in acute, in vitro models. According to basic ligand/receptor relations, receptor expression is generally downregulated when ligands are overexpressed chronically, however, this notion is complicated by the very location and nature of TAARs. It is generally accepted that TAAR1 is primarily located intracellularly [177]. How this unique behaviour translates to altered receptor expression is still unknown. Lastly, TAAR reportedly undergoes heterodimerization, which may result in biased signalling outputs [19]. Indeed, it has been reported that this heterodimerization modulates the signalling capacities of GPCRs, thereby altering their sensitivity for ligands [63, 202]. Putative candidates include adrenergic, serotoninergic, dopaminergic and glutaminergic receptors, and as such, signal modulation by receptor pairs is largely underestimated, especially considering the vast expression of GPCRs in any one cell [189]. Interestingly, GPCR distribution and expression is dependent on several factors, including gender and disease condition. To add to the complexity, intermediate receptor pairings have also been suggested [212], further complicating pharmacological and drug discovery studies. As such, Berry et al. [18] suggested targeting GPCR dimers, which should mitigate undesired side effects and increase ligand selectivity.

These data suggest that TAAR1 may have a modulatory (down-regulatory) role in trace aminergic signalling, but that this effect may be dependent on nature of receptors with which heterodimers are formed upon ligand binding. Given the clear role for TA in IBS aetiology and symptomology, elucidation of the potential of TAAR as therapeutic target is high priority.

**Fig. 2:**
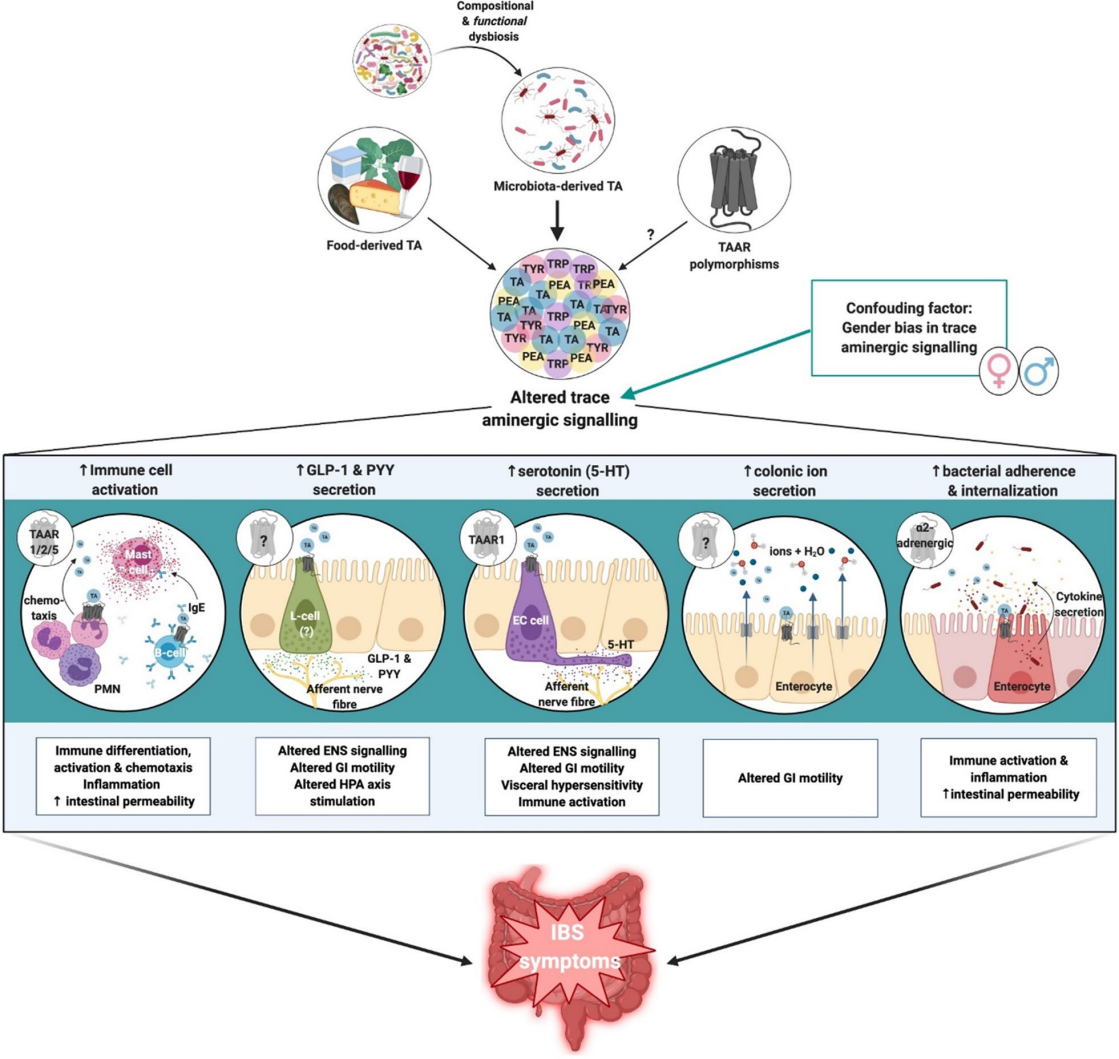
Altered trace aminergic homeostasis as a potential aetiological factor in IBS pathogenesis. The predominant risk factor promoting altered trace aminergic signalling in the GIT is functional microbial dysbiosis, which varies TA load. This altered signalling is gender dependent, and results in functional consequences, which manifest as clinical IBS symptoms. *TA* trace amine, *TAAR* trace amine associated receptor, *IgE* immunoglobulin E, *IL-4* interleukin 4, *GLP-1* glucagon-like peptide-1, *PYY* peptide tyrosine tyrosine, *HPA* hypothalamic pituitary axis, *5-HT* serotonin, *ENS* enteric nervous system, *GI* gastrointestinal, *PMN* polymorphonuclear cells

The importance of the trace aminergic system as a multipronged role player, which effects many sites, is visually represented in Fig. 2. This includes a summary of the known functions of TA in the GIT, and the plausible links to IBS symptomology.

### Is trace aminergic signalling gender dependent?

Turning attention back now to the female predominance in IBS, it is important to consider whether trace aminergic signalling also shows gender-dependence, as this may impact on not only drug discovery, but also patient management.

Indeed, with the emergence of the concept “microgenderome”, researchers have shown that the microbiome is both shaped by reproductive hormones and that the microbes in turn are able to regulate levels of these hormones [76]. An example of this is prevalent when one considers the more specific “estrobolome” [119, 183], which collectively encompasses intestinal microbes (or rather their gene repertoire) capable of producing estrogen metabolizing enzymes (such as β-glucuronidase). In healthy individuals the actions of the estrobolome increases intestinal reabsorption of estrogens, while in dysbiotic conditions this is reduced [9]. Similarly, the microgenderome can also modulate 5-HT signaling (as discussed earlier) and interestingly again do so via estrogens. As such, the effect that altered trace aminergic signalling could have on 5-HT release and action, could be predetermined by lifelong exposure to and priming by E2.

Furthermore, TAAR signalling is differentially activated by distinct TA profiles in different genders. Indeed, in a study by Liberles and Buck [126] murine TAAR5 was reported to respond strongly to extremely diluted urine from male mice, but not female mice or prepubescent males. Notably, three ligands identified for murine TAARs (mTAARs) are natural components of mouse urine: PEA, isoamylamine and TMA, which act as ligands for mTAAR4, mTAAR3 and mTAAR5 respectively, of which isoamylamine and TMA are enriched in male *vs* female mouse urine [83, 161]. Furthermore, isoamylamine in male urine is reported to act as a pheromone, fast-tracking the onset of puberty in female mice [161]. Thus, by utilizing mTAAR5, mice could theoretically determine the gender and sexual status of other mice, which suggest that at least some mTAARs detect social cues [94, 163] that may stimulate certain behaviours or physiological responses. While this study has not been replicated in humans, we are of the opinion that altered trace aminergic signalling (as reported in modern chronic diseases) may be the result of altered gender dynamics. In modern society, with the rise of female emancipation and modern hygiene practices, both females and males may have inadvertently altered their ‘social cues’. This of course remains to be confirmed, as this may have far-reaching implications for disease preventative strategies in IBS-high risk populations. Nevertheless, the little available data suggest that amount and distribution of at least some TA within a host are gender (or even sexual status) specific, which may have implications for diseases in which onset parallels the onset of puberty, such as IBS.

Another link between gender and altered TA homeostasis is chronic psychological stress. According to several studies, elevated PEA levels in urine correlated with increased stress and stress response, in both humans and rodents [65, 92, 174, 213]. Typically, it was thought that women are more vulnerable to life stress [208, 241], and are more prone to depression, anxiety and somatization than men [2, 25, 49]. Women also seem to present with exaggerated IBS symptoms when stressed [98]. In addition, gender-related differences in the prevalence of depression becomes apparent after menarche and continued until peri-menopause [93], which parallels IBS symptom peak onset. These studies, along with several more recent studies, report evidence for sexual dimorphism in stress response in the context of IBS [110, 111, 225]. However, the link between chronic stress and increased urinary PEA levels is less clear, although limited research to date does again point to an estrogen link in this context. Interestingly, in a study investigating the relationship of urinary PEA levels and personality traits (MMPI) in healthy individuals, reported that males had lower PEA levels [151], a finding that had previously also been reported [182]. Although concrete mechanisms and IBS specific data is lacking, current data supports an interpretation in favour of a gender dependence in trace aminergic signalling. As such, fluctuations of especially female reproductive hormones may alter trace aminergic signalling. Given the comprehensive body of literature that already exist on female reproductive hormone replacement therapy, it may be possible to expand on the manipulation of hormone levels for therapeutic effect in IBS. This option warrants further research in this context.

## Where to from here?

From the literature reviewed here, both female reproductive hormones (especially E2) and TA potentially modulate EC cell functioning. While no studies have directly explored the role of an altered trace aminergic system in patients with IBS, it is eluded to. The fact that TAARs are present in almost all leukocyte populations, and the supply of significant amounts of their ligands (TA) originate from food and gut microbiota, suggests that the trace aminergic system is conveniently positioned at the interface of diet/nutrition, gut microbiome, and mucosal immunity, all of which are implicated as aetiological factors in IBS pathogenesis. In terms of proposed mechanisms, dyshomeostasis of the trace aminergic system may result in altered colonic ion secretion, hyperreactivity of the immune system and fluctuations of 5-HT levels causing aberrant 5-HT signalling. While disruption of trace aminergic homeostasis may occur due to TAAR polymorphisms or increased TA supply due to changes in diet, functional microbial dysbiosis seems to be the predominant risk factor. Aberrant trace aminergic functioning can result in altered leukocyte differentiation, activation and chemotaxis, all while microbes more efficiently adhere to and infiltrate intestinal epithelium. The ensuing pro-inflammatory state of the gut could manifest in the symptomology clinically prevalent in IBS. As such, we suggest that microbial-derived TA (and the functional consequences perpetuated by the trace aminergic system) should be considered aetiological factors in the pathogenesis of IBS. Furthermore, since an altered trace aminergic system results in fluctuations of intestinal 5-HT, which is already targeted for modulation by current medications for IBS, then it is feasible to suggest that TAARs be considered targets for future therapeutic action, with the specific focus of reducing oxidative stress and inflammation.

## Conclusions

In conclusion, the microgenderome concept may explain, at least in part, the gender bias observed in many chronic inflammatory conditions. The notion of host-intrinsic factors, which are reinforced and manipulated by commensal bacteria, could underpin the relationship between an altered trace aminergic homeostasis and female predominance in IBS. In order to elucidate the nature of relationship between the trace aminergic system and reproductive hormones, specifically E2, and their influence on IBS, areas of overlap, such as modulation of serotonin and ion secretion and susceptibility to oxidative stress and inflammation requires further investigation.

## Data Availability

Not applicable.

## Abbreviations

IBS: Irritable bowel syndrome
IBD: Inflammatory bowel disease
GI: Gastrointestinal
GIT: Gastrointestinal tract
ERs: Estrogen receptors
MDA: Malondialdehyde
5-HT: Serotonin/5-hydroxytryptamine
EC: Enterochromaffin
E2: 17β-Estradiol
SERT: Serotonin reuptake transporter
Prog: Progesterone
SIBO: Small intestinal bacterial overgrowth
TA: Trace amines
PEA: β-Phenylethylamine
TRP: Tryptamine
TYR: ρ-Tyramine
AADC: Aromatic l-amino acid decarboxylases
TAAR: Trace amine associated receptors
LAB: Lactic acid bacteria
MAOI: Monoamine oxidase inhibitor
NE: Norepinephrine
TMAO: Trimethylamine *N*-oxide
TMA: Trimethylamine
T_1_AM: 3-Iodothyronamine
PMN: Polymorphonuclear cells
GLP-1: Glucagon-like peptide-1
PYY: Peptide tyrosine tyrosine
IgE: Immunoglobulin E
